# Biodegradation of Choline NTF_2_ by *Pantoea agglomerans* in Different Osmolarity. Characterization and Environmental Implications of the Produced Exopolysaccharide

**DOI:** 10.3390/polym15193974

**Published:** 2023-10-03

**Authors:** Abrusci Concepción, Amils Ricardo, Sánchez-León Enrique

**Affiliations:** 1 Departamento de Biología Molecular, Facultad de Ciencias, Universidad Autónoma de Madrid, UAM, Cantoblanco, 28049 Madrid, Spainenrique.sanchezleon@estudiante.uam.es (S.-L.E.); 2 Centro de Biología Molecular Severo Ochoa, CSIC-UAM, 28049 Madrid, Spain

**Keywords:** bacteria, ionic liquid, toxicity, emulsifying, antioxidant

## Abstract

A specific microorganism, *Pantoea agglomerans* uam8, was isolated from the ionic liquid (IL) Choline NTF_2_ and identified by molecular biology. A biodegradation study was performed at osmolarity conditions (0.2, 0.6, 1.0 M). These had an important influence on the growth of the strain, exopolysaccharide (EPS) production, and biodegradation (1303 mg/L max production and 80% biodegradation at 0.6 M). These conditions also had an important influence on the morphology of the strain and its EPSs, but not in the chemical composition. The EPS (glucose, mannose and galactose (6:0.5:2)) produced at 0.6 M was further characterized using different techniques. The obtained EPSs presented important differences in the behavior of the emulsifying activity for vegetable oils (olive (86%), sunflower (56%) and coconut (90%)) and hydrocarbons (diesel (62%), hexane (60%)), and were compared with commercial emulsifiers. The EPS produced at 0.6 M had the highest emulsifying activity overall. This EPS did not show cytotoxicity against the tested cell line (<20%) and presented great advantages as an antioxidant (1,1-diphenyl-2-picryl-hydrazyl radical (DPPH) (85%), hydroxyl radical (OH) (99%), superoxide anion (O_2_^−^) (94%), chelator (54%), and antimicrobial product (15 mm). The osmolarity conditions directly affected the capacity of the strain to biodegrade IL and the subsequently produced EPS. Furthermore, the EPS produced at 0.6 M has potential for environmental applications, such as the removal of hazardous materials by emulsification, whilst resulting in positive health effects such as antioxidant activity and non-toxicity.

## 1. Introduction

Great industrial advances have allowed the development of many anthropogenic products with applications in many fields including industrial, agrochemical, pharmaceutical, food, and biotechnological [1,2,3]. These products are not exempt from causing serious problems for the environment and public health [4,5,6,7]. Ionic liquids (ILs) are a group of anthropogenic compounds that have been classed as “green” chemistry [8,9,10]. They are salts that, due to the nature of their composition, are formed by organic cations and a wide variety of organic and inorganic anions, allowing an infinite number of these compounds. Their exceptional properties, such as chemical and thermal stability, ionic conductivity, low vapor pressure, and high solvent power, among others, have made them an alternative to conventional solvents [11,12,13]. However, ionic liquids are not exempt from being able to cause problems in the environment [14,15]. This sparked a wave of new designs in the field of ionic liquids, the so-called third generation ionic liquids, intended to be safer for the environment where they were going to be implemented. Among these ionic liquids, those based on Choline should be highlighted. Their most desirable attributes, compared with other types of ionic liquids, were undoubtedly their biodegradable properties. Furthermore, they could be used in industrial processes such as CO_2_ absorption [16,17], and very promisingly, in biotechnology [18,19,20]. This, together with their low price, contributed decisively to the expansion of their usage. However, there are large gaps in the understanding of this type of choline-based ionic liquids, both in terms of their behavior in the aquatic environment and their cytotoxicity, since these are not yet extensively researched [21,22].

One strategy that can be used for the elimination of ionic liquids is bioremediation processes. The use of allochthonous microorganisms with the capacity to decontaminate these environments is the most environmentally friendly alternative. The use of microorganisms has, therefore, the advantage of being ecological, efficient, and economically very profitable. Previous work focused on the biodegradation of ILs [23,24,25] but not in the isolation of specific microorganisms from these compounds. Nor has the importance of the exopolysaccharides (EPSs) produced through IL biodegradation processes been deeply studied [26]. Therefore, there is a need to explore how these polymers can influence the environment where they are produced and their impact on the environment and public health [27,28]. The hypothesis is that the EPSs produced by specific microorganisms from the biodegradation of Choline NTF_2_ under certain environmental conditions can directly influence the possible capacities of the EPSs by varying their emulsifying activity, chelating, and antioxidant activity, among others. The specific objectives of this research were the isolation and identification of specific microorganisms, the biodegradation of IL in different osmolarities and understanding the influence of the chemical composition, morphology, toxicity, and environmental relevance of the EPSs obtained. For this, a series of investigations were carried out to test this hypothesis. The strain *Pantoea agglomerans* uam8 was identified, the biodegradation of the IL was monitored through the production of CO_2_ and the analysis of ion residues, and the EPS was characterized with different analytical techniques (transmission electron microscopy (TEM), scanning electronic microscopy (SEM), matrix-assisted laser desorption/ionization/time-of-flight (MALDI TOF), gas chromatography (GC–MS), high performance liquid chromatography–tandem mass spectrometry (HPLC–MS/MS), attenuated total reflectance/FT-infrared spectroscopy (ATR FTIR), thermogravimetric (TGA), and differential scanning calorimetric (DSC)). Furthermore, the applications of the EPSs were also studied including their bioremediation capabilities by monitoring their emulsifying activity and chelation properties. Additionally, cytotoxicity, morphology, reactive oxygen species and antimicrobial activity of the EPS were studied at a cellular level.

## 2. Materials and Methods

### 2.1. Materials

#### 2.1.1. Chemicals

The compounds were supplied by different companies. The (2-Hydroxyethyl) trimethylammonium bis(trifluoromethylsulfonyl)imide Iolitec 99% Choline NTF_2_ (IL) was obtained from IoLiTec Ionic Liquids Technologies GmbH (Heilbronn, Germany); the olive, sunflower, sesame, and coconut oils from Hipercor (Madrid, Spain); sephadex G-100 column from Aldrich Chemical Company, Inc. (Milwaukee, WI, USA); and trifluoroacetic acid (TFA) from Aldrich^®^ (Schnelldorf, Germany). The following were purchased from Sigma-Aldrich (Madrid, Spain): diesel, hexane, toluene, dextrans standard, 1,1-diphenyl-2-picrylPolymers hydrazyl radical (DPPH), hydrogen peroxide (H_2_O_2_) 30%, salicylic acid 99%, pyrogallol, hydrochloric acid (HCl) 37%, potassium hydroxide (KOH), phosphate-buffered saline (PBS), hydrochloric acid (HCl) 37%, polyoxyethylene sorbitan monolaurate (Tween 20), sodium dodecyl sulphate (SDS), and 2-[4-(2,4,4-trimethylpentan-2-yl) phenoxy]ethanol (Triton X-100).

#### 2.1.2. Microorganisms, Growth Assay, Cell Line and Storage

Trypticase soya agar (TSA), Luria–Bertani (LB), Dulbecco’s modified Eagle’s medium (DMEM) fetal bovine serum (FBS), L-glutamine, penicillin, streptomycin, catalase kit, and MTT (3-[4,5-dimethyl-thiazol-2-yl]-2,5-diphenyltetrazolium bromide) kit were obtained from Sigma-Aldrich (Madrid, Spain). *Escherichia coli* (*E. coli* CECT 516), *Staphylococcus aureus* (*S. aureus* CECT 8753), *Enterococcus faecalis* (*E. faecalis* CECT 184), *Pseudomonas aeruginosa* (*P. aeruginosa* CECT 108), and *Salmonella typhimurium* (*S. thyphimurium* CECT 409) were obtained from Colección Española de Cultivos (Spain). API 20E was obtained from BioMerieux España S.A. The HeLa cell line was purchased from CLS (Cell Line Service, Eppelheim, Germany). The UltraClean microbial DNA isolation kit was obtained from MO BIO Labs., Inc. (Solana Beach, CA, USA), and DNA purification JetQuick kit from Genomed (Leesburg, VA, USA).

### 2.2. Isolation and Identification of the Microorganism

A medium was prepared as described in Abrusci et al. [29] (MGM), g/L: K_2_HPO_4_ 0.5, KH_2_PO_4_ 0.04, NaCl 0.1, CaCl_2_ 2H_2_O 0.002, (NH_4_) 2SO_4_ 0.2, MgSO_4_ 7H_2_O 0.02, and FeSO_4_ 0.001, and supplemented with glucose and Choline NTF_2_ compound as a carbon source at the concentrations of 4 g/L and 50 mg/L, respectively. The media were exposed in the Universidad Autónoma de Madrid (Spain), with orientation 40°32′32.8″ N/3°41′31.0″ W for 72 h. Every 24 h, samples of 100 µL were taken and cultured in trypticase soya agar (TSA) and grown at 30 °C to isolate microorganisms. After the preliminary morphological and biochemical characterization, as used by Abrusci et al. [30], bacterial strains were identified using commercial identification kits. A commercial assay was employed: API 20E. The preparation and inoculation procedures followed the recommendations of the manufacturer with the strains being incubated at 30 °C for 24 h. The strain uam8 was amplified using polymerase chain reaction (PCR) and identified as undertaken by Morro et al. [31]. The universal 16 S rRNA gene primers 27 F (5′-AGAGTTTGATCCTGGCTCAG-3′) and 1492 R (5′-GGTTACCTTGTTACGACTT-3′) were used for PCR. The obtained sequences were compared with the sequences in NCBI GenBank using the Basic Local Aligment Search Tool (BLAST) (https://blast.ncbi.nlm.nih.gov/Blast.cgi). The selected sequences were aligned with CLUSTAL X [32].

### 2.3. Aerobic Biodegradation, Bacterial Growth, Microbial Cells, and pH. Analysis of Ion Residues Choline NTF_2_

The aerobic biodegradation of the compound as a carbon source was prepared following the method described in Section 2.2, and the addition of NaCl (0.2 M/11.08 g/L, 0.6 M/35 g/L and 1.0 M/58.44 g/L) to strain uam8 was performed at 30 °C by indirect impedance measurements. The bioassays have been previously described in the literature [33]. These were performed in bioreactors filled with 1.5 mL of bacterial suspension. These were placed in plastic materials with 1.5 mL of 2 g/L of the aqueous KOH solution. The impedance was averaged on a Bac-Trac 4300 apparatus (SY-LAB Geräte GmbH). The percentage of biodegradation was calculated as a percentage of the ratio between the cumulative amount of CO_2_ produced in the biodegradation at time, t, and the theoretical amount of carbon dioxide. Formula (1):

% Biodegradation = ([CO_2_]Prod/[CO_2_]Theor.) × 100. (1)

Bacterial growth was monitored with a spectrophotometer Biowave II+ (Biochrom Ltd.) to an optical density of 550 nm (OD_550_ nm). Microbial cells were evaluated as a colony-forming unit (CFU) by different dilution plating incubated at 30 °C for 72 h with TSA agar medium. A Thermo Orion pH Meter (model, 2 Star) was used to determine the pH values during a period of 72 h [23]. The biodegradation of cation (Choline) and anion (NTF_2_) was determined by high performance liquid chromatography–tandem mass spectrometry (HPLC–MS/MS) using an Agilent Technologies 1100 series—6410B (TQ). An ACE Excel 3 C18-Amide column as a stationary phase was used with a mobile phase of 0.1% formic acid in water. The flow rate was 0.2 mL/min. The temperature for analysis was set at 40 °C. Three independent assays were performed.

### 2.4. Production, Extraction and Purification of Exopolysaccharides

The strain was taken from the stock culture and inoculated in a trypticase soya agar medium (TSA). The plates were incubated at 30 °C for 24 h, after which the strain was transferred directly from the plate into flasks of 100 mL capacity filled with 20 mL of minimal growth medium prepared according to the method described in Section 2.2 plus NaCl (0.2, 0.6 and 1.0 M). The flasks were incubated in a rotary shaker incubator (Biogen) at 30 °C and 110 rpm for 72 h. After the first incubation, 10 mL of this broth (2.5 × 10^7^ cells/mL concentration) was inoculated into flasks of 1000 mL capacity filled with 100 mL of this medium. The flasks were incubated at 30 °C and 110 rpm for 72 h, at which time the stationary phase was reached. Three independent assays were performed. The cultures obtained from the uam8 strain were centrifuged at 13,154× *g* for 30 min at 4 °C Duppont—RC5 [34]. The EPS was precipitated with three volumes of ethanol. This was collected by centrifugation and dissolved in Milli-Q water. Subsequently, it was dialyzed at 4 °C with Milli-Q water for 48 h. Finally, it was lyophilized, and the dry weight was determined. A DEAE-52 anion exchange column (2.6 × 30 cm) was used to determine the purification of the EPS. Deionized water was used for elution and NaCl (0.2–1.5 M) as eluent at a flow rate of 1 mL/min, using the phenol-sulfuric acid method. The fractions were collected and lyophilized, and the EPS was obtained.

### 2.5. Transmission Electron Microscopy (TEM) Analysis and Scanning Electronic Microscopy (SEM)

TEM studies were undertaken to study the influence of Choline NTF_2_ over the uam8 strain [35]. The specimens were prepared by putting ca. 5 μL of a 0.05 wt % solution on a TEM copper grid with carbon support film (200 mesh, CBMSO, Madrid, Spain), and observed using a JEOL JEM-1200 (JEOL GmbH) at 120 kV. All images were recorded digitally with a bottom-mounted 4*4k CMOS camera (TemCam-F416, TVIPS).

Scanning electronic microscopy (SEM) studies were undertaken to investigate the exopolymers produced by the uam8 strain both with and without Choline NTF_2_. The micrographs were obtained using a Philips XL 30 scanning electron microscope operating in a conventional high-vacuum mode at an accelerating voltage of 25 kV. Previously, EPS was coated with a 3 nm thick gold/palladium layer [36].

### 2.6. Exopolysaccharide Molecular Weight and Monosaccharides Identification

The molecular weight of EPS was obtained by matrix-assisted laser desorption/ionization/time-of-flight (MALDI TOF/TOF) analyzer equipped with a Nd:YAG 355-nm laser (Ultraflex III, Bruker) as described previously [34]. Mass spectra were recorded in positive reflector (range 1–10 KDa) and lineal (range 1–20 KDa) modes, using a matrix of 10 mg/mL 2,5-dihydroxibenzoic acid (DHB) in methanol/water (90/10).

Gas chromatography (EVOQ GC–TQ, Bruker) coupled with a mass spectrometry detector (GC-MS) was used to determine monosaccharides following the procedure described in the literature [37]. EPS was hydrolyzed at 120 °C for 2 h with 0.5 M trifluoroacetic acid (TFA). A 1 μL sample with source temperature of 230 °C was injected into the capillary column (30 m × 0.250 mm) and gas helium (1 mL/min). Glucose, arabinose, rhamnose, xylose, mannose, galactose, fructose and sorbose were used as standard. HPLC–MS/MS using an Agilent Technologies 1100 series was used for the determination of amino acids and glucuronic acid [38]. For this, the ACE Excel 3 C18-Amide column (stationary phase) and 0.1% formic acid in water (mobile phase) were used. Flow temperature was 0.2 mL/min at 40 °C.

### 2.7. Attenuated Total Reflectance/FT-Infrared Spectroscopy (ATR FTIR). Thermogravimetric (TGA) and Differential Scanning Calorimetric (DSC) Analysis

The IR spectra of EPS for the determination of the structural functional groups [31] were obtained using a Perkin Elmer BX-FTIR spectrometer coupled with an ATR accessory, MIRacleTM-ATR from PIKE Technologies. Thermogravimetric analysis (TGA) of the EPS (1–3 mg) was performed using a TGA Q-500 (Perkin-Elmer). The heating rate for the dynamic conditions was 10 °C/min, and the nitrogen flow was maintained at a constant rate of 60 mL/min. DSC measurements with the EPS (0.5–2 mg) were performed using a DSC Q100 (TA Instruments). The cuvettes were heated (20 to 600 °C) at a rate of 10 °C/min. Data were analyzed using the TA Universal Analysis software [39].

### 2.8. Emulsifying Activity

The emulsifying capabilities of the exopolymers was evaluated using the procedure described in [40]. The assays were undertaken in transparent cylindrical 5 mL tubes which contained 1.5 mL of an oil phase and 1.5 mL of an aqueous phase. The oil phase contained vegetable oils (olive sunflower, sesame, and coconut) and hydrocarbons (diesel, hexane, toluene). For the aqueous phase, both commercial emulsifying compounds (such as polysorbate (Tween 20), sodium dodecyl sulfate (SDS), Triton X-100), and the obtained EPS were used for comparison purposes. All compounds used had a concentration 3:2 *v*/*v*. The tubes were stirred in a vortex at 2400 rpm for 2 min. After 24, 168 h, the emulsification index (E24, E168) was determined as follows by Formula (2):

E 24 h = HEL/HT × 100(2)

where HEL (mm) is the height of the emulsion layer and HT (mm) is the overall height of the mixture. The emulsion formation and stabilization were also assessed.

### 2.9. Nonenzymatic Antioxidants Assays

Nonenzymatic antioxidants assays for 1,1-diphenyl-2-picryl-hydrazyl radical (DPPH•), hydroxyl radical (•OH), and superoxide anion (O_2_^−^•) were evaluated as indicators of the free radical scavenging activities of the EPS at different concentrations (0.1, 0.25, 1.0, 2.5, 5.0, 7.5 and 10 mg/mL). The tests were carried out following the calculations and the procedure described in Sánchez-León et al. [37]. The DPPH assay was carried out with different concentrations of EPS (50 μL) that were mixed with 100 μL of DPPH (100 μM DPPH–ethanolic solution). The mixture was incubated at 25 °C for 30 min.

The OH assay was performed with the EPS diluted to various concentrations (40 μL) which were mixed with 40 μL of 9 mM ethanol-salicylic acid solution, FeSO_4_ solution (9 mM, 40 μL), and H_2_O_2_ (8.8 mM, 40 μL). The O_2_ assay was effected with different concentrations of EPS (0.3 mL) which were mixed with 2.6 mL of phosphate buffer (50 mM, pH 8.2) and 90 μL of pyrogallol (3 mM), dissolved in HCl (10 mM). Absorbances were measured using a FLUOstar Omega spectrophotometer (DPPH/OD_525 nm_), (OH/OD_510 nm_) and (O_2_^−^/OD_325 nm_). Ascorbic acid (Vc) was used as a positive control.

DPPH/OH Radical Scavenging Activity [%] = [ 1 − (A_1_ − A_2_)/A_0_] *×* 100] (3)

Formula (3) was used to determine the percentage of radical-scavenging activity for DPPH, where A_1_ represents the reaction mixture, A_2_ refers to the reaction mixture without DPPH, and A_0_ denotes the reaction mixture with DPPH and without EPS.

Formula (3) was used to determine the percentage of radical-scavenging activity for OH, where A_1_ represents the reaction mixture, A_2_ refers to the reaction mixture without salicylic acid, and A_0_ denotes the reaction mixture with salicylic acid and without EPS.

Superoxide scavenging activity [%] = 1 − (A_10_/C_10_) − (A_0_/C_0_)] × 100 (4)

Formula (4) was used to determine the percentage of superoxide scavenging activity (O_2_^−^•), where A_0_ and A_10_ represent the reaction mixture at 0 and 10 min, respectively; C_0_ and C_10_ represent the reaction mixture without pyrogallol at 0 and 10 min, respectively.

### 2.10. Toxicity Assay

Toxicity assay was determined using the MTT (3-[4,5-dimethyl-thiazol-2-yl]-2,5-diphenyltetrazolium bromide) test (Sigma Aldrich) on HeLa cells. The toxicity of the EPS was tested at different concentrations (0–400 μg/mL) for 24 h, by the reduction of the MTT reagent to formazan as described by Perez-Blanco et al. [41]. HeLa cells were seeded in a 24-well culture plate (5 × 10^5^ cells/mL). The OD_590 nm_ (FLUOstar Omega spectrophotometer) was recorded using a microplate reader.

### 2.11. Measurement of the Non-Radical H_2_O_2_, Morphological Cell and Catalase (CAT) Assay

#### 2.11.1. Measurement of the Non-Radical H_2_O_2_

##### Damage Inducement

The methodology used to measure the damage of the non-radical H_2_O_2_ on HeLa cells was based on the procedure described by Huang-lin et al. [38]. For this, the cells were seeded at a density of 5 *×* 10^4^ cells per well for 24 h. The medium was removed and replaced with 100 μL of H_2_O_2_ (0.25–2 mM) for 1 h at 37 °C, which was removed after this time. Cell viability was measured using the MTT method as described in Section 2.10.

HeLa cell viability was computed using Formula (5):

Cell viability (%) = (A_1_/A_2_) *×* 100 (5)

where A_1_ represents the absorbance of HeLa cells treated with H_2_O_2_ and the MTT solution, while A_2_ represents the absorbance of HeLa cells that were not subjected to any treatment with the MTT solution.

##### Protective Effect of EPS against Non-Radical H_2_O_2_

The ability of EPS to protect HeLa cells against the non-radical H_2_O_2_ was evaluated following the method described by Huang-lin et al. [38]. For this, the cells were seeded at a density of 5 *×* 10^4^ cells per well for 24 h. DMEM solutions were removed and replaced by EPS diluted in DMEM at different concentrations (25–400 μg/mL) for 1 h. They were removed and 2 mM H_2_O_2_ was added and incubated for 1 h. The MTT method described in Section 2.10 was used to determine cell viability. As a positive control, ascorbic acid (20 mg/mL) was used.

HeLa cell viability was calculated with Formula (6):

Cell viability [%] = (A_1_/A_2_) *×* 100 (6)

where A_1_ refers to cells that were treated with both H_2_O_2_ and EPS, and subsequently exposed to the MTT solution; and A_2_ refers to cells that did not receive any treatment and were exposed to the MTT solution.

#### 2.11.2. Morphological Cell

Cell morphology was determined using an Olympus CKX53 fluorescence microscope with a DAPI filter. The same procedure as that performed with the measurement of the non-radical H_2_O_2_ was carried out. All images were captured with a 10x objective and analyzed with Olympus DP-23 software (Olympus Corporation, JPN).

#### 2.11.3. Catalase (CAT) Assay

The protective capacity of the EPS against oxidative stress was investigated using HeLa cells [42]. A total of 5 *×* 10^5^ HeLa cells were seeded in 24-well plates and incubated for 24 h. The medium was subsequently discarded, and the cells were washed with phosphate-buffered saline (PBS). Then, cells were treated with EPS at various concentrations (50, 100, 200, and 400 μg/mL) that were dissolved in PBS and incubated for 1 h at 37 °C. Once the treatment had elapsed, the supernatants were collected and used for the determination of CAT. This activity was determined using a test kit (Catalase assay Kit from sigma) according to the protocol.

### 2.12. Chelating Activity

The chelating capacity of metals was carried out following the calculations and the operational procedure tested by Huang-lin et al. [38] against ferrous ions using ferric nitrate at two different pH values of the final reaction mixture (2.5 and 5.6). The mixture was prepared with 1.0 mL of EPS (0.1 to 10 mg/mL), 0.05 mL of FeCl_2_ (2 mM), 0.2 mL of ferrozine (5 mM), and 2.75 mL of Milli-Q water and incubated at room temperature for 10 min. Ethylenediaminetetraacetic acid (EDTA) was used as a positive control. An OD_562 nm_ (FLUOstar Omega spectrophotometer) was used.

The chelating ability on ferrous ion was calculated according to Formula (7):

Chelating ability [%] = [(A_0_ − (A_1_ − A_2_)/A_0_)] *×* 100 (7)

where,

A_0_ = OD_562nm_ of the deionized water.
A_1_ = OD_562nm_ of the reaction mixture.
A_2_ = OD_562nm_ of the reaction mixture but without FeCl_2_.

### 2.13. Antibacterial Activity by Agar Well Diffusion Assay

Agar well diffusion assay was used to evaluate the antibacterial activity of EPS following the method reported by Rajoka et al. [43]. The *E. coli* CECT 516, *Staphylococcus aureus* CECT 8753, *Enterococcus faecalis* CECT 184, *Pseudomonas aeruginosa* CECT 108, and *Salmonella typhimurium* CECT 409 strains were used as indicator microorganisms to evaluate the antibacterial activity of EPS. The plates prepared with the Luria–Bertani (LB) medium were inoculated with 100 μL of bacterial suspension (10^7^ CFU/mL). Wells (4 mm in diameter) were made, where 60 mL of EPS was deposited at different concentrations (1–10 mg/mL). The LB agar plates were kept at 4 °C for 1 h and incubated at 30 °C for 24 h. The antimicrobial activity was determined by measuring the diameter of the inhibition zone around the wells.

### 2.14. Statistical Analysis

Analysis of variance test (ANOVA) was performed to undertake the statistical comparisons by using the Statistical Package for the Social Sciences version 21 (SPSS^®^ Inc., Chicago, IL, USA). *p* < 0.05 was considered statistically significant.

## 3. Results and Discussion

### 3.1. Isolation of Bacterial Strain, Morphological and Biochemical Characterization

**Figure 1:**
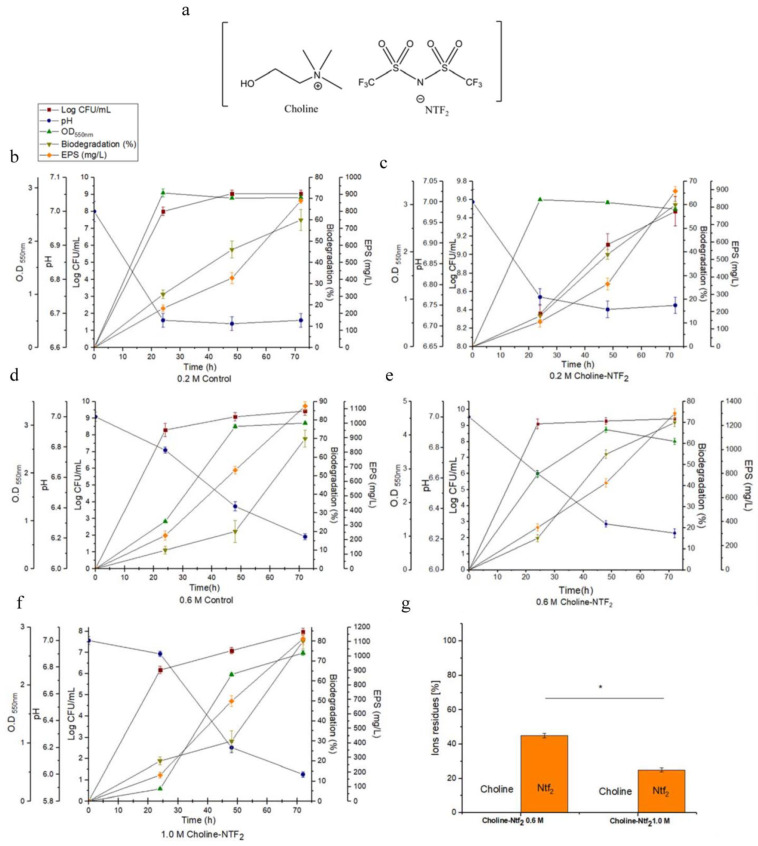
(**a**) Structural representation of the ionic liquid Choline NTF_2_. Optimization of the production of EPS at different saline concentrations and Choline NTF_2_ presence; (**b**) 0.2 M control; (**c**) 0.2 M Choline NTF_2_; (**d**) 0.6 M control; (**e**) 0.6 M Choline NTF_2_; (**f**) 1.0 M Choline NTF_2_; (**g**) detection of ionic liquid (Choline NTF_2_) after biodegradation. * (*p* < 0.05).

The strain, uam8, was isolated as described in Section 2.2 using Choline NTF_2_ as the only carbon source (Figure 1a). The bacterial strain was identified by means of its 16S rDNA sequence obtained after PCR amplification and sequencing. Comparison of the 16S rDNA sequences of the isolated strain with the available sequences in the GenBank database showed that the isolated bacteria was *P. agglomerans* uam8, with a similarity of 100% to MH101508.1. It is a bacterium that belongs to the family of Enterobacteriaceae [44]. Typically, *P. agglomerans* occupies different ecological niches. Therefore, this species is widely distributed in soils, water, plants, animals and humans [45].

The results of the morphological and biochemical tests indicated that the strain *P. agglomerans* uam8 was a Gram-negative bacillus with motility, negative oxidase, positive catalase, and facultative anaerobic characteristics. It did not produce H_2_S and did not hydrolyze the gelatin. It was found to be capable of fermenting a great number of compounds except for sorbitol and inositol and was not sensitive to streptomycin. These morphological and biochemical characteristics are mostly shared among the different strains of *P. agglomerans* [46,47]. In addition to this, even within the different species of the genus, there is a pattern of similarity of these characteristics, such as in the species of *P. vagans* [48], *P.gaviniae* [49], and *P. annatis* [50].

### 3.2. Monitoring of Growth and Biodegradation Parameters. Production of Exopolysaccharides

The experiments (Figure 1) were conducted using both glucose (control) and glucose with an IL (Choline NTF_2_) (Figure 1a) in different osmotic conditions (0.2, 0.6, 1.0 M), during a period of 72 h and at a temperature of 30 °C. The influence that these parameters had on bacterial growth was measured in colony-forming units (CFU), pH, exopolymer production and biodegradation evaluated by indirect impedance measurements. In 0.2 M osmolarity conditions there were no significant differences in growth of *Pantoea agglomerans* uam8 between the control, (0.2 M Control/2.82 ± 0.06) (Figure 1b) and the presence of the IL (0.2 M Choline NTF_2_/2.91 ± 0.04) (Figure 1c) in the studied period. However, with an osmolarity of 0.6 M, the presence of the IL increased bacterial growth (0.6 M Choline NTF_2_/4.168 ± 0.06) (Figure 1e) with respect to the control (0.6 M Control/3.05 ± 0.03) (Figure 1d). With an osmolarity of 1.0 M, the presence of only glucose (1.0 M Control) did not result in any bacterial growth, however this occurred when the IL was present (1.0 M Choline NTF_2_/2.554 ± 0.04) (Figure 1f). In the results, a larger number of CFUs were observed when the IL was present across all studied osmolarities (0.2 M Choline NTF_2_/9.43 ± 0.2), (Figure 1c); (0.6 M Choline NTF_2_/9.47 ± 0.3), (Figure 1e); and (1.0 M Choline NTF_2_/7.98 ± 0.17), (Figure 1f). For 0.6 M Choline NTF_2_ (pH 7 to just 6.4)) (Figure 1e), no acute pH descent was detected, as the acidification of the medium was very low. However, for all other studied conditions, the pH decreased until 6 as was the case of 1.0 M Choline NTF_2_, where the medium was acidified (Figure 1f). pH would be an important factor to take into account in high molarities. It was also observed that the presence of the IL at 0.6 M resulted in the largest production of the exopolymer (0.6 M Choline NTF_2_/1303 mg L^−1^) (Figure 1e), compared with (0.2 M Choline NTF_2_/900 mg L^−1^) (Figure 1c) and (1.0 M Choline NTF_2_/1100 mg L^−1^) (Figure 1f). In the case of *Pantoea sp* [51], high levels of EPS production were associated with environmental stress, caused by high salt concentrations. However, in other cases such as *Volcaniella eurihalina* (0.8 mg L^−1^) [52], *Kocuria rosea* ZJUQH (2008 mg L^−1^) [53], and *Lactobacillus confuses* (25,960 mg L^−1^) [54], the higher production of EPS did not have a direct relationship with high saline concentrations. In our study, this production seems to be associated with the use of the choline cation. The presence of a nitrogen source favored both the growth rate and the production of EPS [55]. The results also indicated that the largest efficiency in the biodegradation of the carbon sources (80%) was reached when both sources (glucose and IL) were available together and at high molarity, at 0.6 M Choline NTF_2_ (Figure 1e) and 1.0 M Choline NTF_2_ (Figure 1f). On the other hand, the analysis of the ions that comprise the IL confirmed that both ions were used by the strain in conditions of high osmolarity (0.6 M Choline NTF_2_ and 1.0 M Choline NTF_2_) (Figure 1g), but in an unequal way. The cation (choline) was not residual, which confirms the use of this by the strain. This indicated that the entry of choline into the cell was carried out successfully, showing that this process was dependent on the salinity of the environment [56]. The entry of choline into the cell could be mediated by ABC-type transporters driven by a proton–sodium gradient. Once inside, the transformation from choline to glycine betaine took place which had an osmoprotective effect on the strain [57]. This favored the adaptation of the strain to saline environments [58]. This adaptation translated into higher rates of cell growth [59] and EPSs production [51]. For the anion (NTF_2_), its biodegradation was independent of the choline cation. Under the conditions of 0.6 M Choline NTF_2_, 42% of the anion was residual, whereas for 1.0 M Choline NTF_2_, only 25% of the anion was residual. These results confirmed a larger utilization by *P. agglomerans* uam8 of the anion (NTF_2_) at a higher osmolarity. This could be because the high concentration of sodium chloride favored the solubility of the anion and its bioavailability. Gram-negative bacteria (*Sphingomonas, Pseudomonas* and *P. agglomenrans*) have been effective in biodegrading toxic compounds such as phospohonium-based ILs [60,61] and chlorinated hydrocarbons (85%) [62].

### 3.3. Transmission Electron Microscopy (TEM) and Scanning Electron Microscopy (SEM)

**Figure 2:**
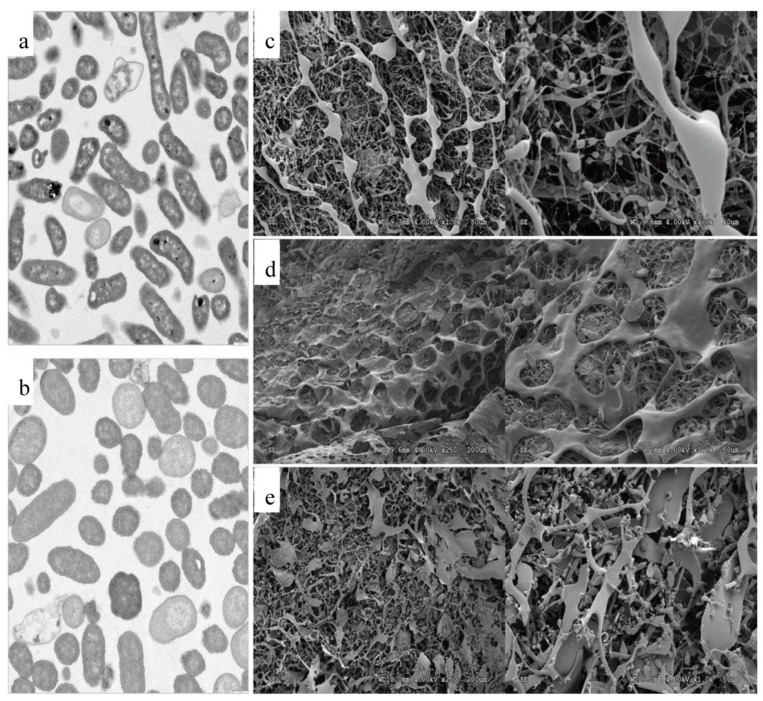
Electron microscopy: (**a**) TEM of cell on 0.6 M Control, (**b**) TEM of cell on 0.6 M Choline NTF_2_, (**c**) SEM of EPS to 0.6 M Control, (**d**) SEM of EPS to 0.6 M Choline NTF_2_, (**e**) SEM of EPS to 1.0 M Choline NTF_2_.

TEM studies were undertaken (Figure 2) to investigate the possible changes that occurred at high osmolarity in bacterial cells both in 0.6 M Control and in the presence of the IL 0.6 M Choline NTF_2_, in the first 24 h. With 0.6 M Control (Figure 2a), the cells presented a contracted cytoplasmic volume with loss of turgor. However, in the 0.6 M Choline NTF_2_ medium, the cells presented a turgid appearance and without any deformations (Figure 2b,c). This revealed that *P. agglomerans* uam8 in the absence of Choline NTF_2_ could not synthesize into glycine betaine through novo synthesis, and the cells were damaged. However, when the bacteria were grown with choline, cell turgor was maintained due to the conversion of Choline NTF_2_ into glycine betaine. This suggests that this strain may have environmental applications in Choline NTF_2_ bioremediation. Similar results were found in *B. subtilis* [63,64], and *Tetragenococcus halophila* [65] which required the presence of choline to produce glycine betaine.

The SEM study revealed that the obtained EPSs at 0.6 M Control (Figure 2d), 0.6 M Choline NTF_2_ (Figure 2e), and 1.0 M Choline NTF_2_ (Figure 2e) presented morphological differences between them. The EPS obtained from 0.6 M Control and 1.0 M Choline NTF_2_ presented a greater number of finely filamentous structures. However, the EPS obtained from 0.6 M Choline NTF_2_ presented broader structures. These differences were due to the osmolarity conditions and the absence or presence of Choline NTF_2_. Similar results were found in the EPSs of *Shewanella oneidensis* strain MR-1 [66] and *Bacillus pseudomycoides* U10 [67]. This indicated that the morphology of the polymers was affected by environmental and nutritional conditions.

### 3.4. Characterization of the Exopolysaccharides

The EPS produced from the biodegradation of 0.6 M Choline NTF_2_ was characterized, because it was under these conditions where the highest EPS production took place and its morphology was the least damaged. In addition, the osmolarity conditions were adjusted to those typical of seawater salinity (35 g/L), which made for more interesting environmental conditions.

**Figure 3:**
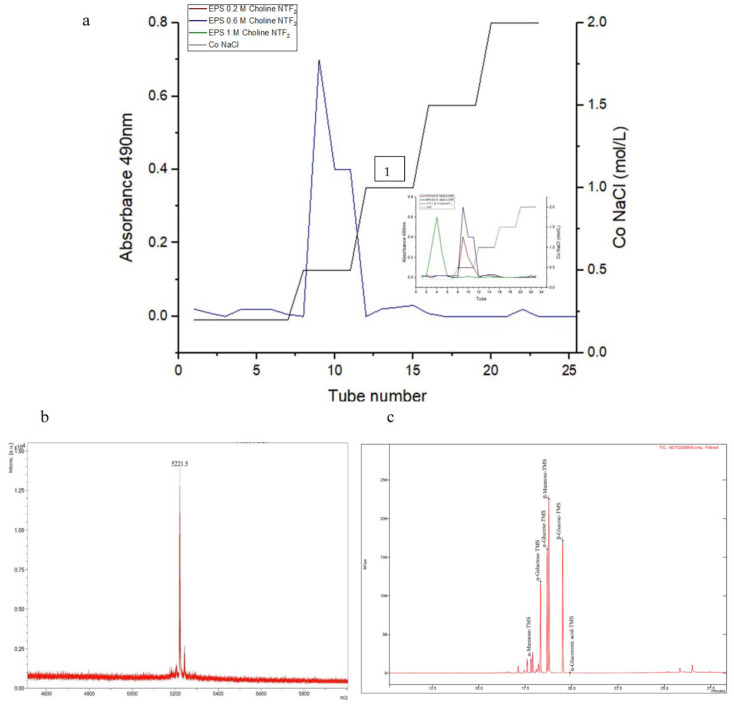
Characterization of the exopolymer EPS extracted from *Pantoea agglomerans*: (**a**) elution curve obtained from the purification of EPS 0.6 M Choline NTF_2_, and (1) of EPS 0.2 M Choline NTF_2_ and 1.0 M Choline NTF_2_; (**b**) MALDI-TOF mass spectroscopy of EPS 0.6 M Choline NTF_2_; (**c**) GC-MS of EPS 0.6 M Choline NTF_2_.

The EPS produced from the biodegradation of 0.6 M Choline NTF_2_ presented an isolated peak, Figure 3a. On the other hand, the other EPSs produced in this study (0.2 M Choline NTF_2_, and 1.0 M Choline NTF_2_) also presented this characteristic. This highlighted that the obtained polymers were pure. This is an important feature of bacterial exopolysaccharides [38]. The mass spectrum of the purified EPS of 0.6 M Choline NTF_2_ showed a molecular weight of 5221.5 Da. (Figure 3b). This low molecular weight that this EPS presented was due to the osmotic conditions. Extreme environmental conditions can give rise to low molecular weight EPSs, as was the case of the EPS produced by *Synechococcus* PCC7942, with a molecular weight of 3–10 KDa [68].

The EPS produced by *P. agglomerans* uam8 from biodegrading 0.6 M Choline NTF_2_ was analyzed by gas chromatography GC–MS Figure 3c. EPS was a heteropolysaccharide, exclusively composed of three monosaccharides, glucose (α–glucose, β–glucose), galactose and α–mannose, with a molar ratio of 6:2:0.5, respectively. The presence of the glucose monomer as a component of these EPSs seems recurrent in the genus *Pantoea*. Preliminary investigations have supported this fact. The *Pantoea agglomerans* strain [69] showed an EPS formed by a heteropolysaccharide that contained mannose and glucose in a ratio of 3:2. This EPS was obtained from glucose and yeast as a carbon source. *Pantoea* sp. (BM39) [51,70] produced an EPS that was a homopolysaccharide, formed exclusively from glucose, from sucrose, glucose and fructose as a carbon source.

Less common monomers such as the heteropolysaccharide of *Pantoea sp*. have also been described in the EPSs of the genus *Pantoea* YU16-S3 [71] with glucose, galactose, N-acetyl galactosamine, and glucosamine in the ratio of 1.9:1:0.4:0.02, respectively. This EPS was obtained from a marine environment, containing peptone and yeast. Some of these clear differences in the compositions of the EPSs can be attributed to genetic and physiological factors.

**Figure 4:**
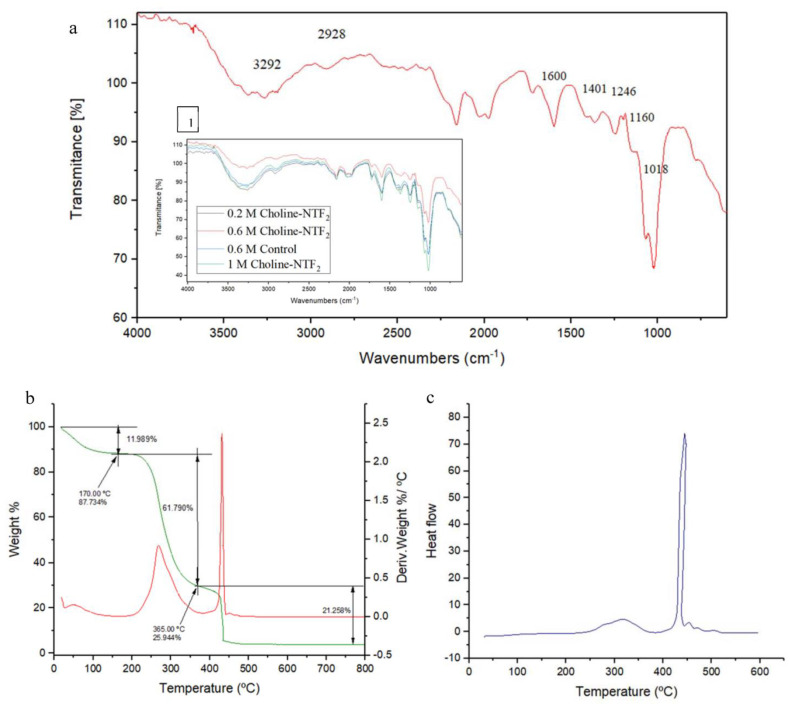
(**a**) ATR−FTIR of EPS 0.6 M Choline NTF_2_ and EPS produced (1) by 0.2 M Choline−NTF_2_, 0.6 M Control and 1.0 M Choline-NTF_2_. (**b**) TGA of EPS 0.6 M Choline NTF_2_. (**c**) DSC of EPS 0.6 M Choline NTF_2_.

FT-IR spectroscopy is an analytical technique that allows the identification of functional groups from absorption peaks [72]. The FT-IR spectrum of EPS from 0.6 M Choline NTF_2_ is shown in Figure 4a. In addition, the FTIR spectra of all the other EPSs produced in this study (0.2 M Choline NTF_2_, 0.6 M Control and 1.0 M Choline NTF_2_), shown in Figure 4a, revealed a broad homology between the EPSs samples. This demonstrated that neither the nutritional nor the environmental factors conditioned the composition of the EPS. The broad and strong peak of 3292 cm^−1^ and 2928 cm^−1^ indicated O-H stretching vibration and C-H stretching vibration, respectively [73,74]. The peak at 1600 cm^−1^ and 1401 cm^−1^ was attributed to the ionic carboxyl group (COO-) and C-H bond vibration, respectively [75]. According to a previous study, the absorption at 1246 cm^−1^ could be attributed to the pyranose ring [37]. Absorption peaks in the 1160 cm^−1^ region indicated C–O–C stretching vibration and the peak at 1018 cm^−1^ was due to C–O–H stretching vibration [76]. FT-IR spectroscopy of the EPS was characteristic of the presence of polysaccharides in the EPS. These groups were also present in the EPS of other strains of *Pantoea agglomerans* [69], and in *Pantoea alhagi* NX-11 [77].

One of the important factors to take into account for an efficient environmental application of bacterial exopolysaccharides is the study of their thermal stability. The TGA analysis showed (Figure 4b) that the EPS (0.6 M Choline NTF_2_ medium) experienced an initial weight loss of 11.04% due to moisture content at approximately 180 °C. This is due to the carboxyl groups of the polysaccharide, which have an affinity for water molecules. Subsequently, a weight loss of around 61% occurred at 365 °C, which was attributed to the degradation of the sample itself. Furthermore, the DSC analysis (Figure 4c) of this sample showed two melting peaks at 317.22 °C and 444.51 °C. The degradation temperature of the purified EPS of 0.6 M Choline NTF_2_ was lower than the purified EPS from *Pantoea* sp. BCCS 001 GH (weight loss (69%) at 318 °C) [78,79] and *Pantoea* sp. YU16-S3 (weight loss (20%) at 200 °C) [71], which contained different monosaccharides in their EPSs. These differences could be decisive in thermal stability and, therefore, in its environmental and industrial application.

### 3.5. Environmental Implications of the Produced Exopolysaccharides

#### 3.5.1. Emulsifying Study

The emulsifying activity of the exopolysaccharides is crucial for its environmental application. This activity is determined by measuring the retention of the emulsion of vegetable oils or hydrocarbons in water after a certain time period. The EPS (0.6 M Choline NTF_2_ medium) emulsification study was carried out at different times (24 h (E24) and 168 h (E168)), concentrations (0.5, 1, and 2 mg/mL), and at pH 7.0, and with both vegetable oils (olive, sunflower, sesame, and coconut) and hydrocarbons (diesel oil, hexane, toluene).

**Figure 5:**
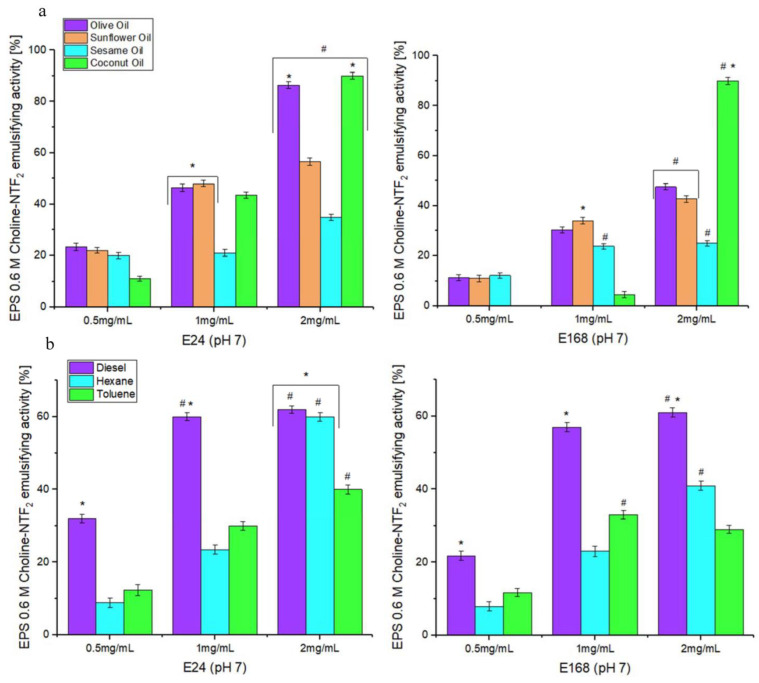
Emulsifying activity of EPS at different concentrations (0.5, 1, 2 mg/mL) and times (E24 y E168): (**a**) emulsifying activity with different natural oils, and (**b**) emulsifying activity with different hydrocarbons. *, statistical differences between different natural oils or hydrocarbons for each concentration (*p* < 0.05). #, statistical differences between different concentrations (*p* < 0.05).

Figure 5a shows the effectiveness of EPS as an emulsifier for the studied vegetable oils. The results indicated that for 24 and 168 h, the effectiveness of EPS as an emulsifier of vegetable oils was not effective at the concentrations of 0.5 and 1 mg/mL. Emulsification percentages of more than 50% were not reached for any of them. In the concentration of 2 mg, for 24 h (*p* < 0.05), high emulsification values were given for olive oil ((E24) 86.4%), sunflower oil ((E24) 56%), and coconut oil ((E24) 90%). However, for 168 h, the activity was only maintained for coconut oil ((E168) 90%). Figure 5b shows the effectiveness of EPS as an emulsifier for hydrocarbons. The emulsification was effective at 1 mg/mL only for diesel ((E24) 60%), and at 2 mg/mL for diesel ((E24) 62%) and hexane ((E24) 60%). For 168 h, the emulsifying activity was maintained only for diesel, in concentrations of 1 mg/mL and 2 mg/mL ((E168) 57%), ((E168) 61%)), respectively.

The results indicated that EPS was very efficient at 2 mg/mL compared with the results obtained by *Pantoea* sp. [78], which required higher concentrations of EPS (5 mg/mL) to emulsify olive oil ((E24) 58.9%), sunflower oil ((E24) 52.9%), and hexane ((E24) 62%). These differences in the emulsifying capacity of the different EPSs could be due to different factors such as their chemical composition and morphology [80].

**Figure 6:**
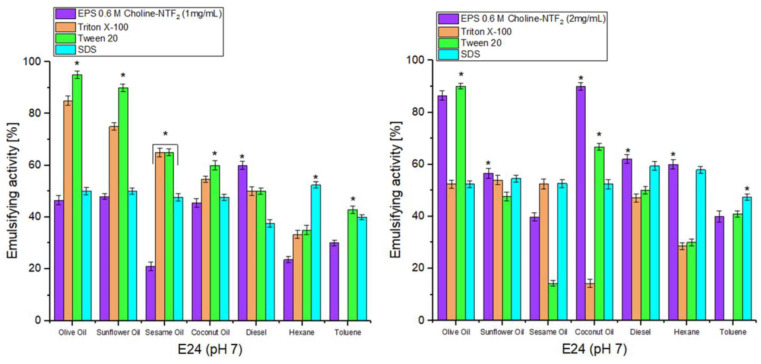
Comparison of emulsifying activity at different EPS 0.6 M Choline NTF_2_ concentrations (1, 2 mg/mL) against commercial emulsifiers (Triton X-100, Tween 20 and SDS) across different natural oils and hydrocarbons. * (*p* < 0.05).

In order to see the environmental advantages of using natural polymers over commercial compounds, the EPS was also tested with three commercial emulsifiers (Triton X-100, Tween 20, and SDS), Figure 6.

The results indicated that at a concentration of 1 mg, the EPS only presented a better emulsifying (*p* < 0.05) activity for diesel ((E24) 60%) compared with commercial emulsifiers (Triton X-100 ((E24) 50%), Tween 20 ((E24) 50%) and SDS ((E24) 37.5%)). However, for a concentration of 2 mg, the emulsifying activity of the EPS was significantly (*p* < 0.05) improved. This increased for olive oil ((E24) 86.4%), sunflower ((E24) 56.5%), coconut ((E24) 90%), diesel ((E24) 62%), and hexane (E24) 60%), compared with commercial emulsifiers. For the latter, only Tween 20 obtained a very slight improvement for olive oil ((E24) 90%), only 4% more when compared with EPS.

Natural EPSs have the advantage of not presenting unwanted side-effects which can be present in commercial emulsifiers [81,82,83]. They are more respectful to the environment for any industrial application.

**Figure 7:**
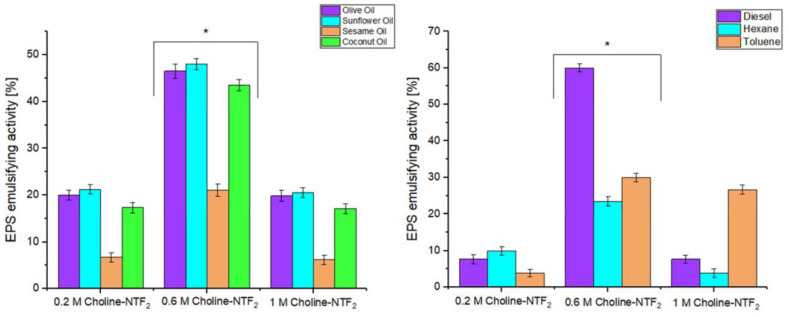
EPSs emulsifying activity of 0.2 M Choline NTF_2_, 0.6 M Choline NTF_2_ and 1.0 M Choline NTF_2_ with different natural oils and hydrocarbons. * (*p* < 0.05).

The emulsifying activity study was also carried out with the other EPSs produced from biodegrading different concentrations of Choline NTF_2_ (0.2 and 1.0 Choline NTF_2_), at a concentration of 1 mg. This is shown in Figure 7, where the EPSs produced from 0.2, 0.6, and 1.0 M Choline NTF_2_ are compared. The results indicated that for the vegetable oils and hydrocarbons, the EPSs of 0.2 M, and 1.0 M Choline NTF_2_ did not have a notable emulsifying activity, with values that did not reach 20% in most cases. However, the EPS obtained from 0.6 M Choline NTF_2_, presented 47% higher activity than the other EPSs for vegetable oils, and even higher in hydrocarbons, with a 60% increase in emulsifying activity for 0.6 M Choline NTF_2_. This indicated that the morphology of the EPS had a decisive role in this activity [37].

#### 3.5.2. Nonenzymatic Antioxidant, Toxicity, Non-Radical H_2_O_2,_ Morphological Cell and Catalase (CAT) Assay

**Figure 8:**
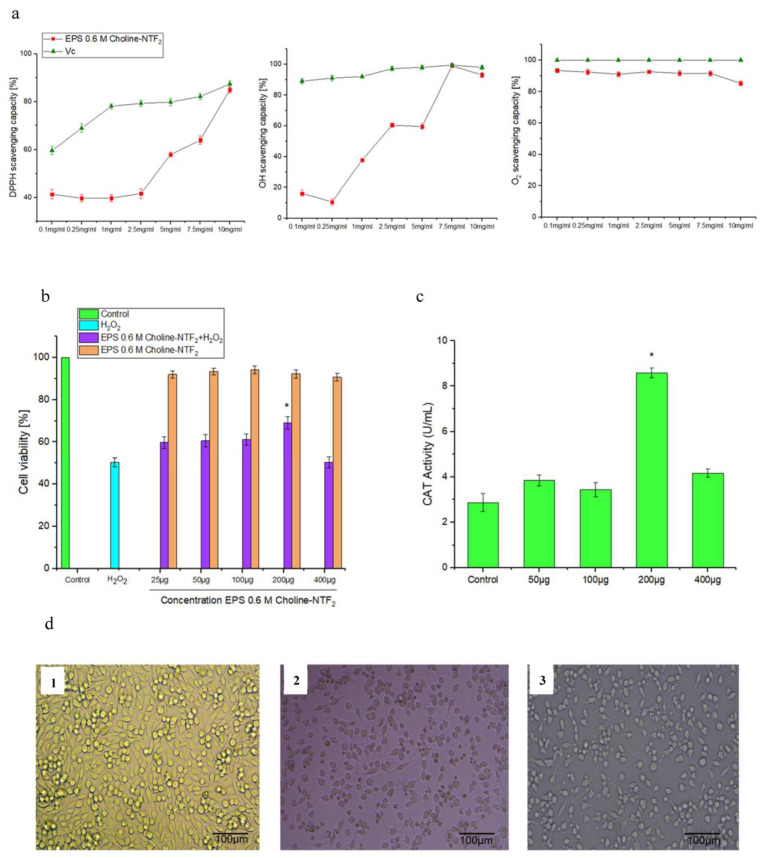
(**a**) Antioxidant effect of EPS 0.6 M Choline NTF_2_ with DPPH, hydroxyl (OH) and superoxide radical. (**b**) Biocompatibility of EPS 0.6 M Choline NTF_2_ and evaluation of the H_2_O_2_-damaged HeLa cells protection. (**c**) Catalase activity by the presence of EPS 0.6 M Choline NTF_2_. (**d**) Effects of EPS 0.6 M Choline NTF_2_ on morphological alterations of H_2_O_2_-damaged HeLa cells. Control was untreated HeLa cells, (1), incubated with H_2_O_2_ (2 mM) (2), pretreated with of EPS 0.6 M Choline NTF_2_ (200 μg/mL) (3). Cells were photographed under phase contrast microscopy bar 30 μm. * (*p* < 0.05).

Nonenzymatic antioxidant assays as shown in Figure 8a (radical scavenging activity (DPPH), hydroxyl radical (HO•), and superoxide scavenging activities (O_2_^−^)) have been used as instruments to explore the antioxidant activity of exopolysaccharides [84].

The antioxidant capacity of the EPS obtained from 0.6 M Choline NTF_2_ was tested in the range of 0.1 to 10 mg/mL, for all assays. For radical scavenging activity DPPH, Figure 8a, EPS presented a high scavenging activity of 85 ± 1.3% versus 87.4 ± 1.4% of VC at 10 mg/mL. In the case of the radical scavenging activity for the hydroxyl radical (HO•), of the EPS (Figure 8a), the results indicated that the EPS had an excellent capacity, with 99 ± 1.3%, thus, obtaining the same capacity as the control, named VC (99.5 ± 1.3%), at 7.5 mg/mL. For superoxide scavenging activities (O_2_^-^), shown in Figure 8a, EPS had a scavenging capability of 94.3 ± 1.3% compared with VC with a scavenging capability of 100% at a concentration of only 0.1 mg/mL. The EPS presented an efficient scavenging activity compared with other EPSs obtained by different species such as *Tetragenococcus halophilus* SNTH-8 with DPPH and hydroxyl radicals activity of 63.53% and 50.19%, respectively, at 12 mg/mL [85]. *Rhodococcus qingshengii* QDR4-2 resulted in DPPH, hydroxyl and superoxide radicals of 49.09 ± 1.54%, 30.24 ± 2.82%, and 39.18 ± 2.70%, respectively, at 3 mg/mL [86]. These results indicated that EPS was very efficient for nonenzymatic antioxidants under these environmental conditions.

The cytotoxicity results showed that EPS (0.6 M Choline NTF_2_ medium), with all the tested concentrations, maintained cell viability, above 90% and very close to control Figure 8b. The results were not significantly different (*p* < 0.05). EPS did not cause damage to the tested cell line. Studies carried out in this same genus revealed that for the EPS of *Pantoea* sp. YU16-S3 [71], proliferation increased at a concentration greater than 100 µg/mL in the NIH3T3 cell line tested. This absence of cytotoxicity, in 0.6 M Choline NTF_2_, in a wide range of concentrations, gives EPS a great advantage for environmental and public health applications.

Cell viability and morphological study was carried out to evaluate the possible effect of a non- radical H_2_O_2_. HeLa cells were highly sensitive to H_2_O_2_ injury, Figure 8b. A marked decrease in cell viability was shown when cells were exposed to 2 mM H_2_O_2_. This meant a reduction in viability of 50.3%, Figure 8b. In addition to this, the morphology of the cells was affected at this concentration (Figure 8b). H_2_O_2_ interfered with the development of filipodia, unlike control cells, where these were well developed. These results indicated that an accumulation of reactive oxygen species (ROS) had occurred [38].

On the other hand, cells previously incubated with EPS exhibited a morphology typical of control cells, with good development of filipodia, Figure 8(c1), at a concentration of 200 μg/mL. Treatment with EPS at that concentration, Figure 8(c3), was found to protect against H_2_O_2_-induced damage with a viability of more than 70%. This could be directly related to the morphology of the EPS (Figure 2), where it could achieve a critical scaffold structure, and this could be necessary to induce protection [34]. The reducing power of the EPS was due to its remarkable radical scavenging capacity and endowed it with potent antioxidant activity. In addition to this, an enzymatic antioxidant assay was conducted to determine the catalase (CAT), Figure 8d. EPS increased CAT activity in HeLa cells, at all concentrations tested. However, the highest CAT activity was 8.54 ± 2.9 U/mL in the presence of EPS, at a concentration of 200 μg/mL, compared with the value of 2.56 ± 2.1 U/mL of the control (*p* < 0.05). The increase in the CAT activity by the EPS allowed the prevention of the formation of hydroxyl radicals and stimulated the enzymatic defence of the cells [87]. The ability of other EPSs to protect against this cell damage has been described in *Lactobacillus kimchi* SR8 (8.78 ± 0.31 U/mL at 50 mg/Kg) [88] and in other genera such as *Lactococcus lactis* (8.19 ± 1.36 U/mL at 40 mg/Kg) [89]. This reveals that EPS can effectively act as an antioxidant preventing lipid peroxidation of the membrane.

#### 3.5.3. Chelating Activity and Antibacterial Activity

**Figure 9:**
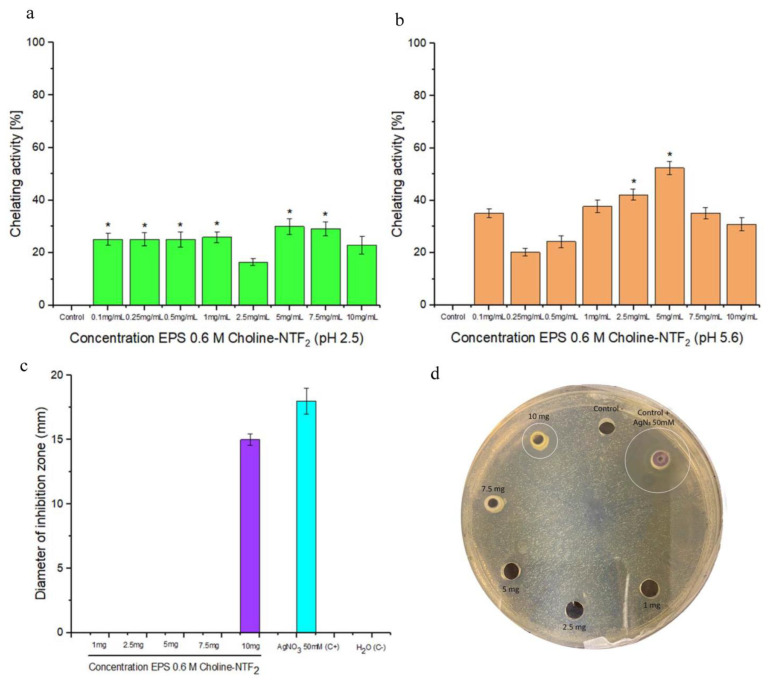
Chelating activity at different concentrations of EPS 0.6 M Choline NTF_2_ measured at pH (**a**) 5.6 and (**b**) 2.5. (**c**) Diameter of inhibition zone of EPS 0.6 M Choline NTF_2_ on *Staphylococcus aureus*. (**d**) Image of the inhibition halo of EPS 0.6 M Choline NTF_2_. * *p*< 0.05.

EPS obtained from the biodegradation of 0.6 M Choline NTF_2_ presented an iron chelation (Figure 9a,b) capacity of 42.2% and 52.4% at 2.5 and 5 mg/mL, respectively, for pH 5.6. These results demonstrated that EPS was better than the EPSs previously isolated from *Bacillus amyloliquefaciens*, as it exhibited a chelating capacity on Fe^2+^ at 2 mg/mL of up to 30.5% [90]. The chelating capacity of the polymer contributed to preventing the formation of free radicals. This was due to the presence in the exopolysaccharide of specific functional groups (OH and COO^−^) [91]. Furthermore, under limiting environmental conditions, the EPS appears as a good candidate in bioremediation processes.

On the other hand, the results of the antimicrobial activity of EPS showed a strong zone of inhibition with a halo over 15 mm where the bacterial growth was inhibited for one of the tested strains. EPS was able to prevent the growth of *Staphylococcus aureus* CECT 8753 (15.0 ± 0.4 mm), Figure 9c,d, at the highest concentration tested. Different studies demonstrate the antimicrobial capacity of EPSs from marine bacteria with the capacity to inhibit bacterial pathogens [92]. This antimicrobial activity could be directly related to the chelation capacity of EPS. This could hinder the absorption of nutrients and vital processes of synthesis of genetic material [43,93].

## 4. Conclusions

*Pantoea. agglomerans* uam8 isolated from ionic liquid Choline NTF_2_ is presented as a good candidate for its biodegradation under saline stress conditions. This study provides valuable information on the behavior of the exopolysaccharides produced from the biodegradation of an ionic liquid. Varying the osmolarity conditions of the medium had an effect on the morphology of the produced exopolysaccharides whilst the chemical composition remained unchanged. The exopolysaccharide produced in 0.6 M conditions had the highest emulsifying activity for natural oils and hydrocarbons compared with those produced in different osmolarities. The 0.6 M Choline NTF_2_ exopolysaccharide did not present cytotoxicity and had antioxidant capabilities. Furthermore, this EPS had other important biotechnological advantages, such as chelating and antimicrobial properties. The role of these polymers produced by bacteria in biodegradation processes opens up an important avenue of research, to glimpse how they can influence the environment where they are produced, and their direct relationship with the environment.

## Data Availability

Not available.
